# Exploring Sexual Well-being in Infertile Couples: A Gender Perspective

**DOI:** 10.1192/j.eurpsy.2025.2306

**Published:** 2025-08-26

**Authors:** K. Mahfoudh, S. Hamzaoui, F. Askri, A. Ouertani, U. Ouali, A. Aissa, R. Jomli

**Affiliations:** 1 Department A, Razi Hospital, Manouba, Tunisia

## Abstract

**Introduction:**

Infertility can have a profound impact on couples, causing emotional distress and negatively affecting sexual well-being. In Tunisia, it contributes to over 20% of divorce cases due to relationship strain. Despite its significance, research on the influence of infertility on sexual experiences, particularly gender differences, remains limited.

**Objectives:**

This study aims to evaluate the effects of infertility on sexual health of Tunisian infertile couples and to compare these effects between men and women.

**Methods:**

We conducted a cross-sectional study involving sexually active infertile couples who had been under follow-up for at least one year at a specialized Assisted Reproductive Technology center in Tunis. Each participant completed closed-ended questions regarding their sexual experiences following the infertility diagnosis, including the frequency of sexual intercourse, preferred types of sexual activities, sexual positions believed to enhance conception, and overall sexual rhythm. The Arizona Sexual Experiences Scale (ASEX) was used in Arabic to assess sexual function.

**Results:**

A total of 60 infertile couples participated in the study. The average age of women was 35.07 ± 4 years while the average age of men 41.1 ± 6 years. Regarding sexual intercourse frequency, 35% of women (n=21) and 27% of men (n=16) reported a decrease, with no significant gender difference (p=0.426).

Infertility did not significantly alter preferences for sexual practices, as 78% of women (n=47) and 85% of men (n=51) reported no changes. Vaginal penetration was the predominant activity for both sexes (100%), while mutual masturbation was engaged in by 68% of women and 72% of men. Oral sex was reported by 57% of women and 53% of men, with no significant gender differences (p>0.05).

In terms of sexual positions, 48% of women and 50% of men favored specific positions to enhance conception, with no significant differences (p=0.995). However, 48% of women and 64% of men adhered to a calendar-based rhythm, with women perceiving this regimen as more detrimental to spontaneity (p=0.038 and p=0.041).

Sexual dysfunctions were significantly more common in women, with a prevalence of 28% compared to only 5% in men. Desire disorders were the most commonly reported sexual dysfunction for both genders. Women exhibited significantly higher rates of physical and psychological arousal problems, as well as orgasmic disorders (p<0.05).

**Conclusions:**

Screening for sexual dysfunction in infertile couples is essential not only for improving sexual health but also for providing tailored psychological support that considers gender differences. By identifying and addressing these issues, healthcare providers can enhance the overall well-being of couples dealing with infertility challenges.

**Disclosure of Interest:**

None Declared

